# Gene expression profiles associated with cigarette smoking and moist snuff consumption

**DOI:** 10.1186/s12864-017-3565-1

**Published:** 2017-02-14

**Authors:** Subhashini Arimilli, Behrouz Madahian, Peter Chen, Kristin Marano, G. L. Prasad

**Affiliations:** 1 RAI Services Company, PO Box 1487, Winston-Salem, NC 27102 USA; 2 Department of Microbiology and Immunology, Wake Forest University School of Medicine, Winston-Salem, NC 27101 USA; 3 Quire Inc., 20 S. Dudley St., Memphis, TN 38103 USA; 4 RAI Services Company, 401 North Main Street, Winston-Salem, NC 27101 USA

**Keywords:** Gene expression, Biomarkers, Smoking, Moist snuff, qRT-PCR, PBMC

## Abstract

**Background:**

Among the different tobacco products that are available on the US market, cigarette smoking is shown to be the most harmful and the effects of cigarette smoking have been well studied. US epidemiological studies indicate that non-combustible tobacco products are less harmful than smoking and yet very limited biological and mechanistic information is available on the effects of these alternative tobacco products. For the first time, we characterized gene expression profiling in PBMCs from moist snuff consumers (MSC), compared with that from consumers of cigarettes (SMK) and non-tobacco consumers (NTC).

**Results:**

Microarray analysis identified 100 differentially expressed genes (DEGs) between the SMK and NTC groups and 46 DEGs between SMK and MSC groups. However, we found no significant differences in gene expression between MSC and NTC. Both hierarchical clustering and principle component analysis revealed that MSC and NTC expression profiles were more similar than to SMK. Random forest classification identified a subset of DEGs which predicted SMK from either NTC or MSC with high accuracy (AUC 0.98).

**Conclusions:**

PMBC gene expression profiles of NTC and MSC are similar to each other, while SMK exhibit distinct profiles with alterations in immune related pathways. In addition to discovering several biomarkers, these studies support further understanding of the biological effects of different tobacco products.

**Trial registration:**

ClinicalTrials.gov. Identifier: NCT01923402. Date of Registration: August 14, 2013. Study was retrospectively registered.

**Electronic supplementary material:**

The online version of this article (doi:10.1186/s12864-017-3565-1) contains supplementary material, which is available to authorized users.

## Background

The long-term health consequences of cigarette smoking have been well documented [1]. For example, cigarette smoking is a major risk factor for lung cancer, Chronic Obstructive Pulmonary Disease (COPD) and Cardiovascular Diseases (CVD), and smokers experience higher rates of mortality relative to non-smokers for these disease states [1, 2]. Cigarette smoking is known to exert local (lung and buccal cavity) and systemic effects, and hence adversely impacts multiple organ systems. Smoking-induced oxidative stress and inflammation are hypothesized as key mechanisms that drive smoking induced diseases [2]. Smoking has been known to alter key signaling pathways and suppress immune responses, among other physiological processes [2]. At cellular and molecular levels, chronic smoking induces a wide range of macromolecular and biochemical changes. For example, several investigators have identified differentially expressed genes in several organ/tissue systems, including lung [3], nasal epithelia [4], buccal cells [4] and peripheral blood mononcuclear cells (PBMCs) [5–7] in smokers. Genes affected by cigarette smoke include those involved in cell survival, inflammation, tumor suppression, and apoptosis and are implicated in smoking-related diseases [8].

Smokeless Tobacco Products (STPs) are a diverse category of tobacco products that are consumed worldwide. Consumption of STPs may be associated with an increased risk for oral and other cancers as well as increased risk of mortality from ischemic heart diseases, depending on the type of product usage [9]. Fermented moist snuff, or dipping tobacco, is the widely consumed oral STP in the US [10]. Existing US epidemiological data suggests moist snuff consumption is generally associated with reduced health risks, relative to smoking, although risk for certain CVD mortality is elevated compared to non-consumers of tobacco [9, 11].

Findings from epidemiological studies among cigarette smokers and smokeless tobacco users in the US indicate that relative to never tobacco use, smokeless tobacco use has been associated with less mortality than cigarette smoking. In particular, data from the American Cancer Society’s Cancer Prevention Study II (CPS-II) indicate, among male smokeless tobacco users compared with male never users of tobacco, the adjusted risks of mortality (i.e., hazard ratios) were 1.28 (95% CI: 0.71-2.32) for chronic obstructive pulmonary disease; 2.00 (95% CI: 1.23-3.24) for lung cancer; 1.26 (95% CI: 1.08-1.47) for coronary heart disease; and 0.90 (95% CI: 0.12-6.71) for oropharynx cancer [12]. In contrast, data from the CPS-II indicate, among male cigarette smokers compared with male never users of tobacco, the adjusted risks of mortality (i.e., hazard ratios) were 10.8 (95% CI: 8.4-13.9) for chronic obstructive pulmonary disease; 21.3 (95% CI: 17.7-25.6) for lung cancer; and 1.9 (95% CI: 1.8-2.1) for coronary heart disease [13]. The relative risk of mortality from oropharynx cancer in this cohort of male cigarette smokers was estimated to be 27.48 (95% CI: 9.96-75.83) [14].

Given the overall burden of disease and mortality due to the consumption of tobacco products, particularly cigarette smoking, harm reduction efforts have led to the recognition of a continuum of risk among different tobacco product categories. Whereas combustible tobacco products such as cigarettes are identified as the most harmful, non-combustible tobacco products are associated with substantially lower harm, relative to the non-consumption of any tobacco as the baseline risk in US [15–17]. Additionally, the available epidemiological data from Sweden among snus (Swedish-type of oral, non-combustible tobacco) supports the reduced risk and harm of non-combustible tobacco relative to cigarettes [18, 19]. Briefly, snus is manufactured from ground air- or sun-cured tobacco and other ingredients using a heat-treatment process that differs from the process of manufacturing of US-style moist snuff [18].

While the harmful effects of cigarette smoking have been extensively investigated and are better understood, the effects of STPs, including moist snuff remain incompletely understood. Previous work from RAI companies evaluated the long-term effects of consumption of moist snuff and cigarette smoking and evaluated several biomarkers of exposure (BioExp) and biomarkers of effect (BioEff) in cross-sectional studies. In the first study, in addition to several BioExp, some biomarkers related to CVD were evaluated [20–22]. A second study, termed biomarker discovery study, focused on BioExp and BioEff in a different study cohort [23, 24]. Although the moist snuff consumer cohort (MSC) in both studies exhibited higher levels of nicotine biomarkers and tobacco specific nitrosamine biomarkers (TSNAs) compared to the smoker cohort (SMK), combustion-related biomarkers in the MSC were comparable to that found in the non-tobacco consumer cohort (NTC) in both studies referenced above. Select BioEff, including those associated with arachidonic acid metabolism, were elevated in both studies in SMK relative to MSC and NTC [22, 23]. Further, global metabolomic evaluation of plasma, saliva and urine collected in the biomarker discovery revealed that SMK exhibit distinct metabolite profiles compared to MSC and NTC cohorts [24]. Overall, BioEff indicative of vitamins C and E, and purine metabolism were altered in SMK, possibly due to increased oxidative stress and inflammatory responses in that cohort, relative to the MSC and NTC [24].

In our continuing efforts to further characterize the physiological changes in long-term smokers and moist snuff consumers, and to identify potential BioEff we have investigated global gene expression changes in the tobacco consumers in the biomarker discovery study cohorts (i.e., SMK, MSC and NTC). Additionally, these studies offer an opportunity to evaluate the concept of risk continuum among tobacco products at a molecular level. PBMCs collected from the study subjects in the biomarker study were utilized in this global gene expression profiling study.

## Methods

### Study design and population

The study design and the cohort characteristics have been previously described [23]. Briefly, this was a single-blind, cross-sectional study of healthy volunteers conducted at the High Point Clinical Trials Center, High Point, NC, USA. The inclusion criteria for cigarette smoker (SMK) group were: males aged 35–60 years; exclusive cigarette smoker of any brand with ≥6 mg “tar”/cigarette by Cambridge Filter Method^1^; consumption of ≥10 cigarettes/day for ≥3 years according to self-report; and expired carbon monoxide (ECO) level 10–100 ppm. The inclusion criteria for moist snuff consumer (MSC) group were: exclusive moist snuff consumer of any brand; consumption of ≥2 cans moist snuff per week for ≥3 years according to self-report; and ECO level 0–5 ppm. The inclusion criteria for non-smoker (NTC) group were: individuals reporting non-use of any tobacco or nicotine-containing products for ≥5 years with an ECO level of 0–5 ppm. Subjects provided written informed consent upon enrollment. The study conformed to ICH Good Clinical Practice guidelines and was conducted according to the principles of the Declaration of Helsinki. The study was approved by a central institutional review board (Independent Investigational Review Board, Inc., Plantation, FL, USA), and registered at ClinicalTrials.gov (ClinicalTrials.gov number: NCT01923402). Additional details on the subject demographics are summarized in Additional file 1: Supplementary Methods.

### Blood sampling, PBMC preparation, and cell type analysis

Blood samples were collected from each subject on the morning of day 1, under fasting conditions and before tobacco consumption. Blood was processed within 2 h of blood collection. Peripheral blood mononuclear cells (PBMCs) were isolated from whole blood as described previously [23, 25]. Blood samples were mixed with Isolymph (CTL Scientific Supply Corp., Deer Park, NY, USA) and incubated for 45 min, and the leukocyte layer was removed and centrifuged at 200 x g for 10 min at room temperature. After removal of the supernatant, the cell pellet was resuspended in PBS at a ratio of PBS:volume of top layer leukocytes drawn of 2:5 mL. Five ml of cell suspension was layered onto 3 mL of Isolymph in 15 mL conical tubes and centrifuged at 400 x g for 45 min at room temperature. PBMC were collected from the middle layer and washed with running buffer (Miltenyi Biotech, Auburn, CA, USA) at 400 x g for 10 min at 4 °C. The isolated cell pellets were dissolved in RLT plus lysis buffer (Qiagen, Valencia, CA, USA) with 1% 2-mercaptoethanol for 30 min on ice before freezing for storage.

After isolating PBMCs from blood by density gradient centrifugation, PBMCs were labeled with different antibodies to measure the distribution of different subsets. Isolated PBMCs (100 μl) were labeled individually with CD2-FITC (Clone RPA-2.10, BD Pharmingen), CD14-FITC (Clone M5E2, BD Pharmingen), CD20-PE (Clone 2H7 BD Pharmingen) and CD56-PE (Clone NCAM16.2, BD Biosciences) for differentiating T cells, monocytes, B cells and NK cells, respectively. After labeling the PBMCs, flow cytometry was performed with BD FACSCalibur (BD Biosciences, San Jose, CA) and by enumerating 10,000 events (cells) per sample. Flow cytometer data was analyzed by using 9.3.1 version of flow Jo software (FlowJo, LLC. Ashland, Oregon). The Tukey–Krammer honest significant difference test (α = 0.05) was used to compare the mean values of the PBMC subtypes among cohorts.

### Microarray experiments

The RNA was prepared from PBMC lysates by SeqWright DNA Technology Services (Houston, TX, USA). Using standard procedures, total RNA was isolated using the Qiagen RNeasy Plus Mini Kit (Qiagen, Valencia, CA, USA), RNA concentrations were determined using a Nanodrop ND-100 spectrophotometer (Thermo Fisher Scientific Inc., Wilmington, DE, USA), and RNA quality was determined by Agilent Bioanalyzer (Agilent Technologies, Santa Clara, CA, USA).

Gene expression profiling was performed using Affymetrix Human Genome U133 Plus 2.0 microarray (Affymetrix, Inc., Santa Clara, CA, USA). The expression of ~47,000 transcripts were analyzed by using 100 ng of RNA sample for double-stranded cDNA synthesis, in vitro transcription of cRNA with biotin labelling and hybridization of cRNA on the microarray. The assay was performed using the Affymetrix standard protocol GeneChip® 3’ IVT Express Kit User Manual (P/N 702646 Rev. 1) and the GeneChip® Expression Analysis Technical Manual With Specific Protocols for Using the GeneChip Hybridization, Wash, and Stain Kit (P/N 702232 Rev. 3).

### Microarray analysis

Microarray data analyses were performed using either Partek’s Genomics Suite Software (Partek, Inc., St. Louis, MO, USA) or the Bioconductor package in R [26]. Prior to analysis, the Partek Batch Remover method was used to remove batch effects caused by variability in reagents, arrays and equipment. All data were normalized using the Robust Multi-array Analysis (RMA) method. Statistical analysis was performed using pair-wise Analysis of Variance (ANOVA) and correcting for type-I error using the False Discovery Rate (FDR) adjustment method [27]. A minimum fold change of greater than 1.25 for up-regulation and less than −1.25 for down-regulation was established as the criteria for differential expression. An FDR adjusted *p*-value of <0.05 was considered statistically significant. Hierarchical clustering and Principal Component Analyses (PCA) were performed using the subset of DEGs identified by pairwise analyses to examine similarities between subjects based on their gene expression profiles. Hierarchical clustering was performed using Ward’s minimum variance method, which aims to find compact spherical clusters by minimizing the within-cluster variance using an optimal value of an objective function (error sum of squares).

Random Forest classification models were built using ‘randomForest’ library in R. For each pair of groups (NTC-SMK, NTC-MSC, and MSC-SMK), the data were randomly divided into equal number of training and test subjects. The Random Forest was built using the training group and the accuracy, sensitivity, and specificity of the model was determined using the test group. We repeated this process 50 times and calculated the average classification accuracy, sensitivity and specificity for the 50 runs. The average Gini importance measure for the 50 runs is reported to indicate the influence of each gene in correctly classifying samples in their appropriate groups. Higher Gini values indicate the relative strength of a particular gene in classifying the samples.

Thomson Reuters (New York, NY, USA) performed pathway analysis on the set genes whose expression levels were significantly affected between SMK group and either MSC or NTC group. This analysis utilized data from the GeneGo Global Network, which contains information on ~24,000 proteins, 2859 compounds, and more than one million interactions. An enrichment analysis was performed, which evaluated the overlap between the differentially-expressed genes and gene groupings from canonical pathway maps (biological mechanism), process networks (metabolic and signaling processes), toxic pathologies, and disease biomarkers.

### Quantitative polymerase chain reaction

To confirm the results obtained by Affymetrix microarray, a TaqMan-based quantitative polymerase chain reaction (qPCR) assay was performed on all 120 subjects in the study. Target genes (*n* = 44) were selected based on the microarray results as well a selected number of genes (AHRR, CCL4L1, CCR2, KLRD1, MAF, S1PR5, SSPN, & TFEC) which are known to be associated with smoking in the literature. A complete list of genes and RT-PCR probes are provided in Additional file 2: Table S1.

The qPCR analysis was performed by SeqWright DNA Technology Services (Houston, TX, USA). Approximately 120 ng (*n* = 118) or 40 ng (*n* = 2) total RNA was digested with DNase I before cDNA synthesis in 10 μL reaction volume using SuperScript VILO cDNA Synthesis kit (Life Technologies, Grand Island, NY, USA). Ten microliters of cDNA reaction mixture from each sample was mixed with 190 μL of nuclease-free water and 200 μL of 2 x Gene Expression Master Mix (Life Technologies, Grand Island, NY, USA), and 100 μL of this mix was loaded into a 384-well The Low Density Array (TLDA) plate. Each sample was loaded into the plate four times. The Applied Biosystems 7900HT Fast System with Software SDS 2.4 (Life Technologies, Grand Island, NY, USA) was used for the qPCR, using the following cycling conditions: stage 1, 50 °C for 2 min; stage 2, 94.5 °C for 10 min; stage 3, 97 °C for 30 s, 59.7 °C for 1 min (40 cycles).

The results of the qPCR analysis were reported as threshold cycle (Ct) values, defined as the fractional cycle number at which the labeled probe emits fluorescence above a fixed threshold. To give a relative ratio of the abundance of the target gene in each sample, the Ct of the target genes were normalized to the Ct of a reference gene (glyceraldehyde 3-phosphate dehydrogenase) using the SDS 2.4, RQ Manager 1.2.1 and DataAssist v3.0.1 software packages (Life Technologies, Grand Island, NY, USA). Statistical significance was determined by paire-wise ANOVA and the *p*-values were adjusted using Benjamini-Hochberg false discovery rate to correct type-I error (false positive).

## Results

### Study population

The characteristics of the patient population have been previously reported [23, 24]. A total of 120 generally healthy subjects completed the study, with 40 subjects in each group (SMK, MSC, and NTC). The majority of patients were Caucasian, with a mean age ranging from 45.0 to 47.2 years. The mean years of product use were 25.1 and 20.6 in the SMK and MSC groups, respectively. During the month prior to the study, the mean number of cigarettes per day consumed by the SMK group was 21.5, while the MSC group consumed a mean 6.3 cans per week of moist snuff.

### PBMC populations in tobacco consumers

**Fig. 1:**
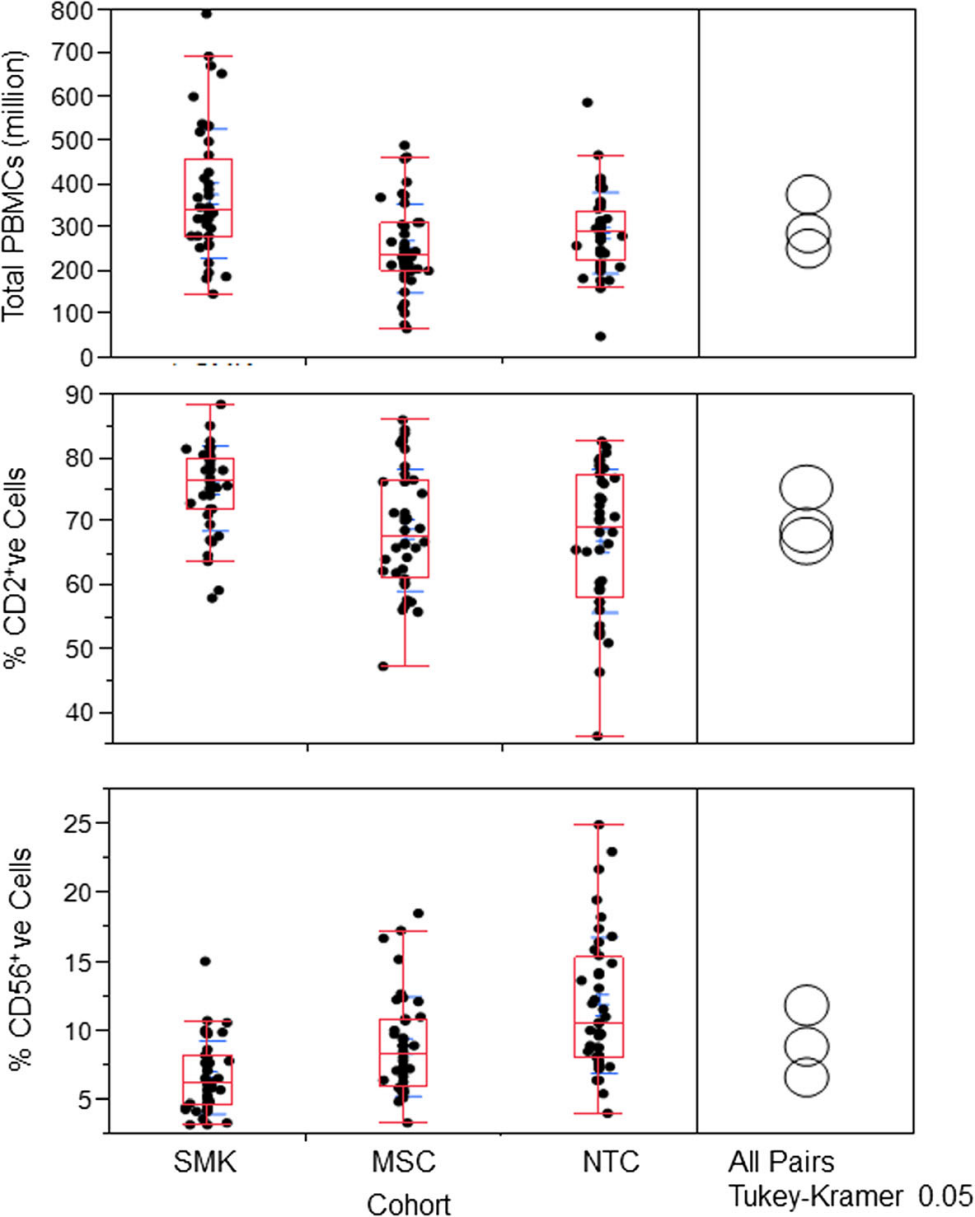
Yield of PBMCs and % positive CD2 and CD56 cells from SMK, MSC and NTC. Yield of total PBMCs from SMK, MSC and NTC cohorts (40 subjects/cohort) are shown on the *top panel*. % CD2 positive cells from SMK, MSC and NTC cohorts are shown in the *middle panel*. % CD56 positive cells from SMK, MSC and NTC cohorts are shown in the *bottom panel*. The Tukey–Krammer honest significant difference test (α = 0.05) was used to compare the mean values among cohorts

In an effort to understand how consumption of combustible and non-combustible tobacco products impacts gene expression in PBMCs, we first examined if there were differences in PBMC levels between the three study groups. Total PBMCs were significantly (α = 0.05, Tukey-Krammer) higher in SMK, relative to MSC and NTC cohorts (Fig. 1). The percentage of CD2^+^ cells (T lymphocytes) in the isolated PBMCs was also significantly higher in SMK, relative to MSC and NTC cohorts. The number of PBMCs or the CD2^+^ cells did not differ significantly between MSC and NTC. In contrast, the average number of CD56^+^ cells (NK cells) was significantly different across all three groups, with NTC group showing the highest followed by MSC and then SMK groups. No differences in monocytes and B lymphocyte populations were detected across the three groups (data not shown). These results suggest that smokers and moist snuff consumers exhibit differences in specific leukocyte subpopulations compared to non-tobacco consumers.

### Gene expression profiling

**Fig. 2:**
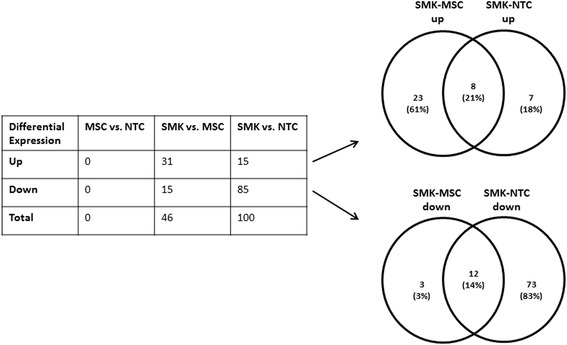
Differentially expressed genes between SMK and either MSC or NTC samples. The number of genes which were significantly (q-value < 0.05) up-regulated or down-regulated in pairwise comparisons are shown for each pairwise comparison. The Venn diagrams show the number of overlapping up-regulated or down-regulated genes for SMK-MSC and SMK-NTC pairwise comparisons

PBMC gene expression levels for 120 subjects were examined using the Affymetrix HG U133 Plus 2 array, which contains probes for over 47,000 human transcripts. Pair-wise statistical analyses were performed between SMK, MSC and NTC groups. Surprisingly, we found no significant (ANOVA, FDR adjusted *p*-value <0.05) differences in gene expression levels between NTC and MSC groups (Fig. 2). In contrast, the expression levels of 100 genes were significantly (ANOVA, FDR adjusted *p*-value <0.05) affected by more than ±1.25 fold between SMK and NTC groups. Notably, 85 out of the 100 genes were downregulated in SMK (Fig. 2; Additional file 3: Table S2). On the other hand, only 46 genes were significantly changed by more than ±1.25 fold between MSC and SMK groups and the majority of the genes (31) were up-regulated in SMK (Fig. 2; Additional file 4: Table S3). Importantly, 20 genes were similarly affected (8 upregulated and 12 downregulated) in both SMK-MSC and SMK-NTC comparisons (Fig. 2).

In general, the magnitudes of the gene expression changes in both comparisons were very small; only five genes were differentially expressed by more than 2-fold. For instance, the expression of IGHA1, GPR15 and LRRN3 was changed by +2.14, +2.13 and +2.07 fold between SMK and MSC, respectively. In addition, the expression of CCL4 and LRRN3 was changed by −2.46 and +2.34 fold between SMK and NTC, respectively. These results are expected since all of the individuals in this study were generally healthy.

Since the magnitudes of the expression changes were small, a total of 44 DEGs were selected for validation by quantitative RT-PCR in the same 120 subjects. Among the 20 DEGs which were similarly affected in both SMK-MSC and SMK-NTC comparisons, only seven DEGs were confirmed in SMK-MSC and 12 DEGs in SMK-NTC comparisons (Table 1). Generally, higher magnitude changes were more likely to be confirmed by qRT-PCR. For instance, all four DEGs with >2.0 fold change were validated by qRT-PCR, whereas only 23% (3 out of 13) and 45% (5 out of 11) DEGs with <1.5 fold change were validated by qRT-PCR in SMK-MSC and SMK-NTC comparisons, respectively (Table 1).

**Table 1:** qRT-PCR validation of 20 DEGs that were similarly affected in SMK-MSC and SMK-NTC pairwise comparisons

		SMK vs. MSC				SMK vs. NTC			
		Microarray		qRT-PCR		Microarray		qRT-PCR	
Gene Symbol	Gene Name	FC	*P*-value (adj.)‡	FC	*P*-value (adj.)‡	FC	*P*-value (adj.)‡	FC	*P*-value (adj.)‡
Upregulated									
GPR15	G protein-coupled receptor 15	**2.13**	**0**	**4.75**	**0**	**2.00**	**0**	**4.27**	**0**
LRRN3	Leucine rich repeat neuronal 3	**2.07**	**0**	**2.18** ^**a**^	**0.0105**	**2.34**	**0**	**2.16** ^**a**^	**0.0069**
SASH1	SAM and SH3 domain containing 1	**1.93**	**0**	**1.74**	**0**	**1.97**	**0**	**1.84**	**0**
ADAMDEC1	ADAM-like decysin 1	1.50	0.002	1.11^a^	0.837	1.45	0.007	1.07^a^	0.8469
PID1	Phosphotyrosine interaction domain containing 1	**1.37**	**0.013**	**1.27** ^**a**^	**0.0037**	**1.31**	**0.004**	**1.84** ^**a**^	**0**
CLEC10A	C-type lectin domain family 10, member A	**1.35**	**0.013**	**1.29** ^**a**^	**0.0201**	**1.31**	**0.018**	**1.26** ^**a**^	**0.0041**
TMEM45B	Transmembrane protein 45B	1.31	0.047	1.1	0.7332	1.53	0.004	1.41	0.0526
FUCA1	Fucosidase, α-L-1, tissue	1.29	0.022	1.14	0.265	1.29	0.01	1.2	0.0805
Downregulated									
PTGDS	Prostaglandin D2 synthase 21 kDa (brain)	**−1.54**	**0.007**	**−2.18**	**0.0006**	**−1.86**	**0**	**−2.23**	**0**
PRSS23	Serine protease 23	−1.54	0.046	−1.33	0.1248	**−1.98**	**0**	**−1.78**	**0.0152**
KLRB1	Killer cell lectinlike receptor subfamily B, member 1	−1.50	0	−1.79	0.1368	**−1.47**	**0**	**−1.69**	**0.0041**
PTGDR	Prostaglandin D2 receptor (DP)	−1.38	0.04	−1.41	0.265	**−1.55**	**0**	**−1.56**	**0.0391**
FYN	FYN oncogene related to SRC, FGR, YES	−1.38	0.045	−1.29^a^	0.5577	**−1.34**	**0.022**	**−1.18** ^**a**^	**0.029**
NKG7	Natural killer cell group 7 sequence	**−1.36**	**0.046**	**−1.51**	**0.0056**	**−1.57**	**0**	**−1.78**	**0.0001**
GZMA	Granzyme A (granzyme 1, cytotoxic T lymphocyte associated serine esterase 3)	−1.33	0.022	−1.30	0.5038	−1.46	0	−1.50	0.1317
TRD	T cell receptor δ locus	−1.33	0.047	-	-	−1.57	0	-	-
SYTL3	Synaptotagmin-like 3	−1.32	0.047	−1.19	0.1392	−1.28	0.044	−1.13	0.1654
SEPT2	Septin 2	−1.30	0.034	−1.08	0.837	−1.26	0.033	−1.02	0.9315
CACNA2D2	Calcium channel, voltage-dependent, α2/δ subunit	−1.28	0.033	−1.22	0.6041	**−1.48**	**0**	**−2.12**	**0.0423**
GZMM	Granzyme M (lymphocyte metase 1)	−1.26	0.025	−1.10	0.5993	−1.36	0	−1.24	0.0805

Five out of eight up-regulated genes in both SMK-MSC and SMK-NTC comparisons were confirmed by qRT-PCR. Also, seven out of 12 down-regulated genes were confirmed in SMK-NTC. In contrast, only two changes in SMK-MSC were confirmed by qRT-PCR analysis. Bolded values denote microarray changes that were confirmed by qRT-PCR. ^a^denotes multiple qRT-PCR probes were tested but only the best result, with respect to fold change or *p*-value, is reported here. ‡, *p*-values were adjusted by FDR method for microarray experiments and by Benjamini-Hochberg method for qRT-PCR

### Functional analysis of expression data

To gain insights into the molecular and cellular pathways which may be involved in smoking related outcomes, we performed functional analysis on the DEGs to identify enriched pathways, process networks and diseases available in the MetaCore platform. We found that GDNF signaling (*p* < 3.91E-03) and chemotaxis (*p* < 3.53E-04) categories were significantly enriched in the SMK-NTC differentially expressed genes (Table 2). In addition, SMK-NTC differentially expressed genes were highly enriched for pulmonary diseases such as obstructive lung disease (*p* < 2.10E-09), COPD (*p* < 3.68E-08) and asprin induced asthma (*p* < 3.70E-08) as well as vascular skin disease (*p* < 1.13E-08) and hypersensitivity (*p* < 6.14E-09). Importantly, NK cell related inflammation networks (*p* < 1.53E-05) and CD8+ Tc1 cell related to COPD (6.56E-04) pathways were enriched for SMK-NTC differentially expressed genes. In contrast, no disease categories and very few pathways and process networks were found to be significantly enriched for SMK-MSC differentially expressed genes (Additional file 5: Table S4).

**Table 2:** Top pathway maps, process networks and diseases for DEGs in SMK-NTC

Top scoring key ontology terms	*p*-value
Key Pathway Maps	
Role of CD8+ Tc1 cells in COPD	6.56E-04
Development_GDNF signaling	3.91E-03
Generation of cytotoxic CD8+ T cells in COPD	4.24E-03
Development_Role of CDK5 in neuronal development	7.75E-03
Immune resonse_HMGB1/TLR signaling pathway	8.67E-03
Key Process Networks	
Inflammation_NK cell cytotoxicity	1.53E-05
Chemotaxis	3.53E-04
Development_Blood vessel morphogenesis	4.85E-03
Inflammation_Protein C signaling	6.66E-03
Reproduction_Feeding and Neurohormone signaling	1.54E-02
Key Disease	
Lung diseases, Obstructive	2.10E-09
Hypersensitivity	6.14E-09
Skin Disease, Vascular	1.13E-08
Pulmonary Diseases, Chronic Obstructive	3.68E-08
Asthma, Asprin-Inudced	3.70E-08

*P*-values (unadjusted) were calculated using the hypergeometric test. Only the top five categories are reported for each map

### Class prediction

**Fig. 3:**
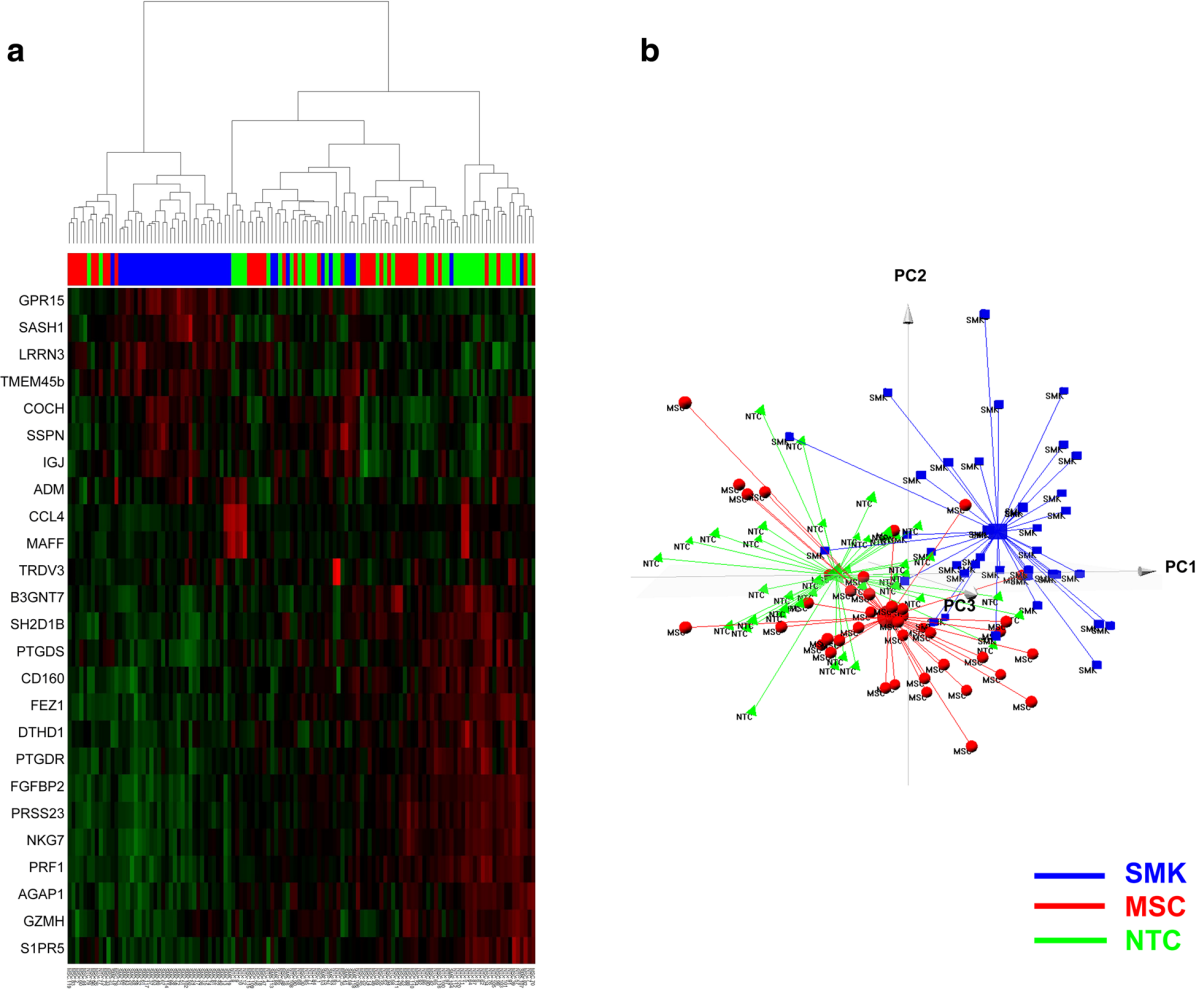
Clustering of 120 subjects based on blood expression profiles of 25 genes which were significantly different by ±1.5 fold between SMK and either MSC or NTC subjects. **a** Hierarchical clustering and heatmap representation of expression values for genes (*rows*) across 120 subjects (*columns*), where low expression is denoted by green and high expression by red. The expression of each gene was normalized across all samples. Subjects were categorized into SMK (*blue*), MSC (*red*), and NTC (*green*). **b** Principal Component Analysis. Subjects were projected according to the first three principal components, which accounted for 35.7% (PC1), 10.0% (PC2) and 6.9% (PC3) of the gene expression variance. Subjects were categorized into SMK (*blue squares*), MSC (*red spheres*), and NTC (*green triangles*). The centroid of each group is depicted by a larger symbol with lines radiating to all of the subjects in that group

To examine if PBMC gene expression profiling could be used to classify individuals into SMK, MSC or NTC groups, we utilized several different approaches. First, hierarchical clustering was performed using all ±1.25 fold differentially expressed genes (Additional file 6: Figure S1). The results showed that individuals in the three groups were interspersed and no clear gene expression pattern could be deduced by visual inspection. This is not surprising since many of the low magnitude changes by pairwise analysis could not be validated by qRT-PCR assays (see above). Therefore, we performed hierarchical clustering using the union of all ±1.50 fold differentially expressed genes, which yielded only 25 unique genes (Fig. 3). At least 29 SMK subjects were clustered together and showed a distinct pattern of up-regulated genes. However, many of the remaining SMK subjects were interspersed with NTC and MSC subjects. In addition, NTC and MSC subjects were intermingled and did not show a clear clustering pattern.

Next, we applied Principal Component Analysis (PCA), which is a robust mathematical method for reducing the dimensionality of the data, to visualize the relationships between subjects based on the variation of the 25 differentially expressed genes across all subjects. When subjects were projected using the first three principal components, which accounted for 52.6% of the total variation, we found that the centroids of the MSC and NTC samples were closer together than to SMK samples respectively (Fig. 3b). These results were consistent with the hierarchical clustering results and revealed a number of SMK subjects whose gene expression profiles were similar to NTC and MSC subjects.

**Fig. 4:**
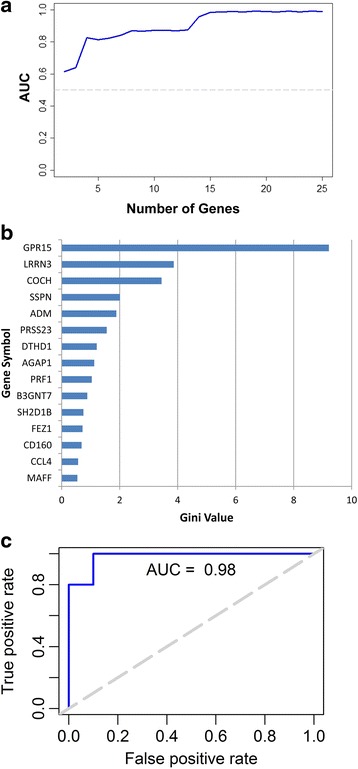
SMK-MSC Random Forest Classification. **a** Average AUC for different number of genes are shown in this figure. The highest AUC of 0.98 is obtained using 15 genes. **b** Average Gini importance of genes across 50 runs of the model indicates the influence of the genes in correct classification (distinction) of SMK and MSC samples. **c** ROC curve associated with the model achieving highest AUC

**Fig. 5:**
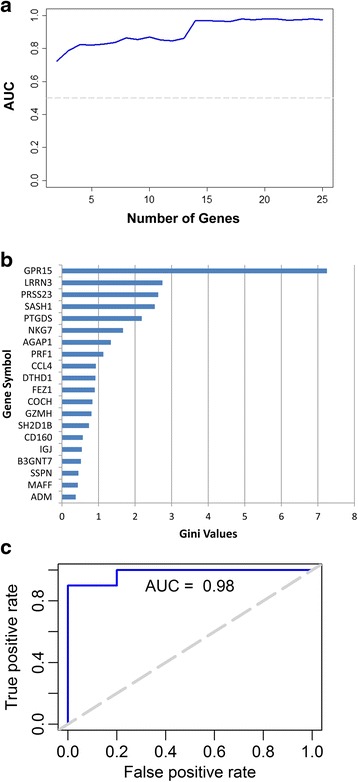
SMK-NTC Random Forest Classification. **a** Average AUC for different number of genes are shown in this figure. The highest AUC of 0.98 is obtained using 20 genes. **b** Average Gini importance of genes across 50 runs of the model indicates the influence of the genes in correct classification (distinction) of SMK and NTC samples. **c** ROC curve associated with the model achieving the highest AUC

Lastly, to perform more rigorous classification analysis, we utilized the Random Forest (RF) method, which is a multivariate classification method based on randomized decision trees. For each pairwise comparison, the RF model was trained on 50% of the subjects in each group and then tested on the remaining subjects. This process was repeated 50 times and the average sensitivity and specificity was calculated. The overall performance of the RF classifier for each pairwise comparison is reported as the Area Under the Curve (AUC) from the Receiver Operator Characteristic (ROC) curve, which displays sensitivity (true positive values) as a function of false positive rate (1-specificity) at various thresholds (Figs. 4 and 5). The highest AUC for both SMK-MSC and SMK-NTC comparisons was 0.98 (Figs. 4a and 5a), which was achieved by using the top 15 and 20 DEGs, respectively (Figs. 4b and 5b).

As a by-product of training the RF classifier, each gene (variable) can be scored with respect to its influence on splitting a sample into different classes, referred to as the “Gini Importance” value. Using this approach, the influence of each gene on the class prediction can be determined, thereby allowing us to identify the most impactful biomarker genes that distinguish between SMK, MSC, and NTC subjects. GPR15 was the best gene predictor of SMK for both MSC and NTC comparison (Gini values of 9.21 and 7.25, respectively). In addition LRRN3 was the second highest predictor, but with substantially lower Gini values in both comparisons (Gini values 3.86 and 2.74). Significantly, all 15 of the top predictor genes in SMK-MSC were also top ranked for predicting SMK from NTC. These results indicate that there is a distinct set of biomarker genes that distinguish SMK from NTC and MSC groups.

## Discussion

In this study, we demonstrated that cigarette smoking results in significant changes in the expression of a small number of genes in PBMCs compared to either moist snuff consumers or people who do not consume tobacco. Notably, this is the first study in which gene expression profiling is conducted in moist snuff consumers. As discussed in the Background section, epidemiological data indicate that US and European smokeless products (snus, in particular) have been associated with reduced risk compared to cigarettes. Consistent with our published work [20, 22–24], which showed that MSC are exposed to reduced levels of combustion-related biomarkers of exposure, MSC exhibited similar gene expression profiles as observed in NTC, but different profiles from SMK. Although the gene expression data are derived from a cross-sectional study, they could support the epidemiological findings in consumers of different classes of tobacco products.

Through Random Forest classification approach we were able to identify a group of genes whose expression levels in PBMCs could accurately (AUC of 0.98) predict SMK from either MSC or NTC groups (Figs. 4 and 5). The top predictor genes for SMK included GPR15, LRRN3, PRSS23, SASH1 and COCH among others. Separately, a 11 gene signature derived from whole blood, consisting of LRRN3, SASH1, PALLD, RGL1, TNFRSF17, CDKN1C, IGJ, RRM2, ID3, SERPING1, and FUCA1 genes was reported to distinguish current smokers from nonsmokers and former smokers [28]. Interestingly, several genes (LRRN3, SASH1 and IGJ) were common to the discriminating set of genes for SMK-NTC comparison (Fig. 5) and the published signature [28].

GPR15 gene encodes a G protein coupled receptor which acts as a chemokine receptor. Consistent with our findings, several studies have shown that GPR15 expression levels and methylation status is associated with smoking and chronic inflammatory pathologies [29–32]. Similarly, the expression and methylation levels of LRRN3 (Leucine rich repeat protein 3) have been associated with smoking in various studies [5, 32, 33]. SASH 1 (SAM and SH3 domain containing 1) expression levels have also been associated with smoking and smoking-related atherosclerosis [28, 34]. SASH1 is believed to be a tumor suppressor in breast and colon cancer and has been shown to inhibit cell migration and enhance cell adhesion of epithelial cells [35]. An increased expression of SASH1 in our study may reflect a host immune response to counteract smoking. The PRSS23 (protease, serine 23) belongs to trypsin family of serine proteases. The PRSS23 gene methylation status is correlated with smoking, but its expression does not appear to be different in the whole blood cells from smokers [36]. Our data, however, show that there is a downregulation of PRSS23 in PBMCs of SMK when compared to NTC and MSC (micro array data), and in SMK vs NTC (RT-PCR) (Table 1). PRSS23 is suggested to regulate cellular proliferation and cancer [37]. In contrast, there is no evidence in the literature which associates COCH (cochlin) with smoking. Thus our studies have identified several established gene expression markers for smoking and have revealed additional marker genes which may provide insights into future smoking research.

Cigarette smoke exposure appears to affect pathways involved in immune response, chemotaxis, as well as inflammatory disorders and lung diseases. Genes involved in stress response or metabolism known to be associated with smoke exposure were not identified in the functional analysis in either the SMK versus MSC comparison or the SMK versus NTC comparison. This suggests that the effect of different categories of tobacco products on molecular pathways could be tissue specific and product category specific. For instance, in airway epithelium and the nasal and oral mucosa, cigarette smoke has been shown to affect pathways involved in inflammation, cell adhesion, tumor suppression, oxidative stress, detoxification, and carcinogen metabolism [3, 4, 38–42]. Studies of the effect of cigarette smoke on monocytes support the results of the present study, with genes related to inflammation, immune response, cell survival, and protein transport affected by cigarette smoke or its constituents [43–45].

Our previous work showed that combustible tobacco product preparations (TPPs) cause DNA damage and are more cytotoxic than non-combustible TPPs [46, 47]. Previous work from us [48] and other researchers [49] has shown that exposure to cigarette smoke constituent phases suppresses several immune responses, such as cytokine secretions in response to stimulation to Toll-like Receptors with agonists, and impairs cytolytic functions of the effector cells in PBMCs. Such compromised immune responses, particularly of NK cells, are hypothesized to contribute to increased susceptibility of smokers to microbial infections and cancer [49]. Further, the levels of perforin, which is an important cytolytic protein, are also suppressed in PBMCs exposed to the constituent phases of cigarette smoke [48]. Consistently, the gene expression results show that PRF1, which codes for perforin, is downregulated in SMK relative to MSC and NTC; interestingly no significant differences were detected between NTC and MSC (Table 3).

**Table 3:** qRT-PCR validation of expression changes that were unique to either SMK-NTC or SMK-MSC comparison

		SMK vs. MSC		SMK vs. NTC		MSC vs. NTC	
Gene Symbol	Gene Name	FC	*P*-value (adj.)‡	FC	*P*-value (adj.)‡	FC	*P*-value (adj.)‡
Unique to SMK vs. NTC							
ADRB2	adrenoceptor beta 2	**−1.44**	**0.0037**	**−1.61**	**0.0003**	−1.11	0.5527
B3GNT7	UDP-GlcNAc:betaGal beta-1,3-N-acetylglucosaminyltransferase 7	−1.13	0.6507	**−1.95**	**0.0358**	−1.73	0.2651
CCL4	chemokine (C-C motif) ligand 4	2.67	0.4409	−7.43	0.0815	−19.84	0.254
CCR2^a^	C-C motif chemokine receptor 2	−1.13	0.648	1.15	0.4312	1.09	0.741
CD160	CD160 molecule	−1.16	0.4317	**−1.64**	**0.0008**	−1.42	0.0572
CST7	cystatin F	**−1.52**	**0.0045**	**−1.71**	**0**	−1.13	0.5229
DTHD1	death domain containing 1	**−1.55**	**0.0221**	**−1.57**	**0.0312**	−1.01	0.9647
ENPP5	ectonucleotide pyrophosphatase/phosphodiesterase 5	−1.03	0.945	−1.41	0.3163	−1.37	0.4482
GPR56	G protein-coupled receptor 56	**−1.73**	**0.0079**	**−1.78**	**0.0002**	−1.02	0.8987
GZMH	granzyme H	**−1.61**	**0.0234**	**−1.96**	**0.0039**	−1.22	0.5751
HHEX	hematopoietically expressed homeobox	1.00	0.9664	1.09	0.5279	1.08	0.7028
KLRD1^a^	killer cell lectin like receptor D1	**−1.36**	**0.0201**	**−1.71**	**0.0001**	−1.26	0.2944
KLRF1	killer cell lectin like receptor F1	1.07	0.9335	−1.34	0.5279	−1.43	0.5229
LPAR6	lysophosphatidic acid receptor 6	**−1.49**	**0.0215**	−1.07	0.7594	1.39	0.2651
MAF^a^	v-maf avian musculoaponeurotic fibrosarcoma oncogene homolog	**1.70**	**0.0215**	−1.30	0.0566	−1.73	0.1712
PRF1	perforin 1	**−1.40**	**0.0091**	**−1.67**	**0.0002**	−1.19	0.4482
S1PR5^a^	sphingosine-1-phosphate receptor 5	1.65	0.0676	**−1.82**	**0.0391**	**−3.00**	**0.0284**
SH2D1B	SH2 domain containing 1B	−1.16	0.648	**−1.70**	**0.0312**	−1.46	0.254
SOCS6	suppressor of cytokine signaling 6	1.05	0.8367	1.12	0.5279	1.06	0.741
TBX21	T-box 21	−1.27	0.0796	**−1.52**	**0.0009**	−1.20	0.2997
TFEC^a^	transcription factor EC	1.34	0.5993	−1.09	0.8409	−1.46	0.5229
TGFBR3	transforming growth factor beta receptor III [	**−1.38**	**0.0079**	**−1.60**	**0.0001**	−1.16	0.4726
Unique to SMK vs. MSC							
ADM	adrenomedullin	**1.66**	**0.0215**	−1.19	0.4309	**−1.98**	**0.0393**
SSPN^a^	sarcospan	−1.71	0.648	−1.30	0.1379	−1.28	0.5164
AHRR^a^	aryl-hydrocarbon receptor repressor	**3.54**	**0.0037**	**3.11**	**0.0041**	−1.14	0.8324

The expression of AHRR gene was also examined since it has previously been linked to smoking. Bolded values denote microarray changes that were confirmed by qRT-PCR. ^a^denotes multiple qRT-PCR probes were tested. Only the best result, with respect to fold change or *p*-value, is reported here. ‡, *p*-values were adjusted by Benjamini-Hochberg method for qRT-PCR

Our qRT-PCR experiments confirmed some well-known complexities associated with global profiling methods in general, and microarray approaches in this particular case. First, not all probes on the microarray may be appropriate for detecting the transcript levels for a given gene. For instance, microarray analysis did not detect a change in aryl hydrocarbon receptor repressor (AHRR) gene between SMK and NTC. AHRR, a well-established repressor and regulator of aryl hydrocarbon receptor (AHR) [50], is widely reported to be hypermethylated in smokers [51]. In contrast to microarray results, our qRT-PCR analysis showed that AHRR transcript was elevated in smokers and was expressed at comparable levels in both SMK-NTC and SMK-MSC comparisons (Table 3). Our finding that smokers exhibit higher levels of AHRR in PBMCs differs from that of other investigators [36] who did not find differences in AHRR transcript levels between whole blood of smokers and non-smokers, perhaps reflecting on the cell types that comprised the starting materials for gene expression analysis. Second, microarray analysis did not reveal any statistically significant differences between MSC and NTC groups after FDR correction. However, qRT-PCR analysis revealed at least two genes (ADM and S1PR) which were significantly changed between SMK and MSC. It is generally recognized that FDR adjustment methods, while necessary to correct for multiple hypothesis testing error, can be too stringent and result in false negatives [27, 52–54].

Several previous studies (reviewed in [51]) have shown that AHRR hypomethylation occurs in smokers, potentially activating aryl hydrocarbon receptor signaling pathway. Consistently, we have found that in buccal cells collected from the same cohort of SMK, several AHRR gene loci were prominently hypomethylated. AHRR methylation is not altered in MSC relative to NTC (manuscript in preparation).

## Conclusions

In summary, in this first genome-wide expression analysis of moist snuff consumers, we found MSC expression profiles are very similar to NTC, while SMK exhibit a distinct gene expression profile. Specifically, previously described markers associated with smokers, such as AHRR, GPR15, LRNN3, COCH and PRSS23 may serve as biomarkers to distinguish different tobacco product consumers.

## Additional files

Additional file 1 Supplementary Methods. (DOCX 18 kb)
Additional file 2: Table S1. qRTPCR probes. (XLSX 15 kb)
Additional file 3: Table S2. SMK-NTC 1.25FC. (XLS 33 kb)
Additional file 4: Table S3. SMK-MSC 1.25FC. (XLSX 16 kb)
Additional file 5: Table S4. Functional Analysis SMK-MSC. (XLSX 10 kB)
Additional file 6: Figure S1. Clustering of 120 subjects based on blood expression profiles which were significantly different by ±1.25 fold between SMK and either MSC or NTC subjects. (A) Hierarchical clustering and heatmap representation of expression values for genes (rows) across 120 subjects (columns), where low expression is denoted by green and high expression by red. The expression of each gene was normalized across all samples. Subjects were categorized into SMK (blue), MSC (red), and NTC (green). (B) Principal Component Analysis. Subjects were projected according to the first three principal components. For additional details, see the caption for Fig. 3. (TIF 4567 kb)

## Abbreviations

DEGs: Differentially expressed genes
MSC: Moist snuff consumers
NTC: Non-tobacco consumers
PBMC: Peripheral blood mononuclear cells
SMK: Smokers
STP: Smokeless tobacco products

## Footnotes

1 Under the Family Smoking Prevention and Tobacco Control Act, FDA has banned the terms “Full Flavor Lights” or “Full Flavor” as a cigarette descriptor as of June 22, 2010. However, at the time of this study, these terms were used (based on tar content/cigarette), thus the “tar” term is included in this manuscript as it accurately reflects the products evaluated prior to such date.
