# Supplementary material for: Gene expression profiles associated with cigarette smoking and moist snuff consumption
Source: BMC Genomics. 2017 Feb 14;18:156. doi: 10.1186/s12864-017-3565-1 (PMC5307792; doi:10.1186/s12864-017-3565-1)
Supplement: Additional file 1 — Supplementary Methods. (DOCX 18 kb) [file 12864_2017_3565_MOESM1_ESM.docx]

GICS-D-16-01461

Gene expression profiles associated with cigarette smoking and moist snuff consumption. Arimilli et al

**Supplementary Methods**

Subject demographics**

The “biomarker discovery study” was a single-blind, cross-sectional study of male tobacco-product consumers and a non-tobacco-consuming control group, conducted at a clinical research unit (CRU), High Point Clinical Trials Center, High Point, NC, USA.

A total of 120 generally healthy male subjects, aged 35–60 years, completed the study in parallel enrolment into one of three groups with the following key inclusion/exclusion criteria: 1) exclusive cigarette smokers (SMK) of any brand ≥6mg “tar” (measured by the Cambridge Filter Pad method), who self-reported smoking at least 10 cigarettes/day for at least 3 years and had an expired carbon monoxide (ECO) level of 10–100 ppm; 2) exclusive MSC of any brand, who self-reported using ≥2 cans of moist snuff/week for at least 3 years and had an ECO of 0–5 ppm; 3) NTC, who self-reported not using any tobacco or nicotine-containing products for at least 5 years and had an ECO of 0–5 ppm. Female subjects were not recruited because of the low rate of ST use in women in the USA.

A majority of the study subjects were Caucasian: 31 in SMK; 35 in MSC and 28 in NTC were Caucasians, and the remaining subjects were of “other” race. The mean ages were: 46.9 years for SMK; 45 years for MSC and 47.2 years for NTC. Based on the self-reports, the SMK smoked for 25.1 years and the MSC used moist snuff for 20.6 years. During the previous month prior to enrolment, the SMK smoked 21.5 cigarettes per day, whereas the MSC used 6.3 cans of moist snuff per week (product use reported as group means). The mean body mass indices were 28.4 for SMK, 29.7 for MSC and 29.4 for NTC.
