# Supplementary figures and images for: Gene expression profiles associated with cigarette smoking and moist snuff consumption
Source: BMC Genomics. 2017 Feb 14;18:156. doi: 10.1186/s12864-017-3565-1 (PMC5307792; doi:10.1186/s12864-017-3565-1)

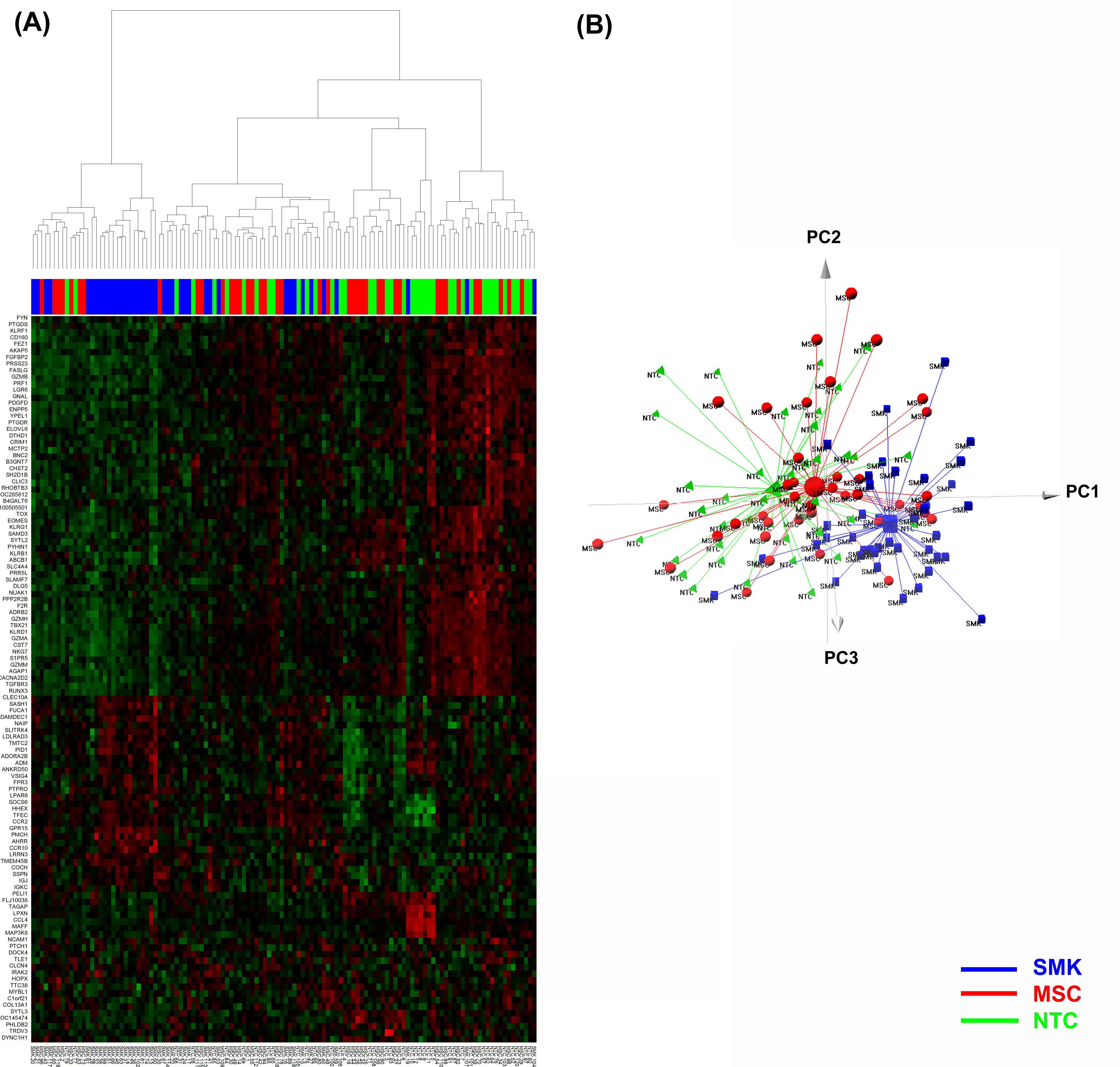

Supplement: Additional file 6: Figure S1. — Clustering of 120 subjects based on blood expression profiles which were significantly different by ±1.25 fold between SMK and either MSC or NTC subjects. (A) Hierarchical clustering and heatmap representation of expression values for genes (rows) across 120 subjects (columns), where low expression is denoted by green and high expression by red. The expression of each gene was normalized across all samples. Subjects were categorized into SMK (blue), MSC (red), and NTC (green). (B) Principal Component Analysis. Subjects were projected according to the first three principal components. For additional details, see the caption for Fig. 3. (TIF 4567 kb) [file 12864_2017_3565_MOESM6_ESM.tif]
